# Crossing experiments suggest a simple genetic basis of complex male self-sacrifice phenotypes in widow spiders

**DOI:** 10.1098/rsbl.2026.0211

**Published:** 2026-08-12

**Authors:** Kardelen Özgün Uludağ, Vladislav Ivanov, Henrik Krehenwinkel, Jutta M. Schneider

**Affiliations:** ^1^ Department of Biology, University of Hamburg, Hamburg, Germany; ^2^ Department of Biogeography, Universität Trier, Trier, Germany; ^3^ Faculty of Mathematics and Natural Sciences, University of Rostock, Rostock, Germany

**Keywords:** *Latrodectus*, monogyny, X-linked inheritance, Mendelian trait

## Abstract

Sexual cannibalism is among the most extreme outcomes of sexual conflict, and the Australian widow spider *Latrodectus hasselti* is well known for this behaviour. Curiously, males of *L. hasselti* actively facilitate cannibalism through self-sacrificial mating traits. During copulation, the male somersaults his abdomen onto the female's fangs and develops an abdominal constriction that prevents his premature death, allowing him to monopolize paternity. The closely related sister species *L. katipo*, however, lacks self-sacrificial mating traits. Given the close relatedness of the two species, the differences in mating traits must have evolved rapidly and are expected to have a rather simple genetic basis. We hypothesize that these two complex traits are tightly linked on the X chromosome and show a single-locus Mendelian inheritance. To test this hypothesis, we backcrossed the two species and scored F2 males for the presence of copulatory somersault and abdominal constriction during mating. Our results suggest that the two traits are not genetically linked, and that the self-sacrificial somersault follows a single-locus X-linked Mendelian inheritance pattern, while the abdominal constriction has a more complex genetic basis. Our work shows that even extremely complex mating traits can evolve rapidly and have a simple genetic basis.

## 1. Introduction

Sexual reproduction has promoted a large diversity of traits that maximize sex-specific reproductive success [1,2]. The frequent divergence of how the two sexes gain maximal fitness has led to the evolution of traits, which benefit the carrier but harm the opposite sex [3]. Indeed, sexual conflict has been identified as a strong evolutionary agent, responsible for sexually antagonistic traits which can be physiological, morphological and behavioural, with well-known examples like traumatic insemination, genital injury and manipulations via seminal fluids [4].

In monogynous mating systems, where males limit themselves to mating with a single female, the intensity of antagonism is predicted to be high [5]. Given the male-biased effective sex ratio in this mating system, a male achieving exclusive paternity with his only mate secures above-average fitness, creating strong selective pressure for the monopolization of paternity [6]. Securing full paternity in an entelegyne spider requires a male to inseminate both spermathecae in two separate copulations using his paired sperm transfer organs (i.e. pedipalps) and plug the spermathecae to prevent further sperm transfer [7]. Plugging is frequently achieved by breaking off the tips of the sperm-transferring embolus on both pedipalps, which prevents further sperm transfer from subsequent males [8]. Such male monopolization strategies create sexual conflict since females have been shown to benefit from polyandry [9]. Hence, females can prevent a male from inseminating and plugging both of her spermathecae by cannibalizing him after the first copulation [5].

A prominent example of sexual cannibalism and counter-adaptive male mating strategies comes from the monogynous Australian redback spider *Latrodectus hasselti*. During the process of sperm transfer, the males invite cannibalism by performing a somersault, which positions the male's posterior opisthosoma onto the female's mouthparts [10]. By tolerating being cannibalized, males achieve longer copulations and higher fertilization success [11]. However, if cannibalism leads to death after the first copulation, males leave the other spermatheca unmated and risk losing 50% of the paternity to another male [12]. A mid-dorsal constriction that arises in the male abdomen during precopulatory courtship can prevent a premature death of the male owing to female-inflicted bites and increase the male's chance of achieving the second and final copulation [13].

Interestingly, females of the closely related sister species *L. katipo* from New Zealand do not engage in sexual cannibalism; males are polygynous and neither show copulatory somersault nor abdominal constriction [10,14,15]. The two sister species hybridize unidirectionally, occurring only between *L. katipo* females and *L. hasselti* males since *L. hasselti* females do not accept *L. katipo* males and respond aggressively to their courtship attempts [10,14]. On the other hand, *L. hasselti* males perform the copulatory somersault consistently, even when mating with *L. katipo* females, which were never observed to cannibalize a somersaulting *L. hasselti* male [10]. These observations suggest that both the somersault and female cannibalistic behaviour in *L. hasselti* have a genetic basis [10].

We hybridized these two widow species to investigate the genetic architecture of the somersault and abdominal constriction. *L. hasselti* and *L. katipo* separated only approximately 100 000 years ago [16], suggesting that these traits evolved rapidly and probably have a simple genetic basis. That even complex behavioural traits can have a simple genetic basis has been shown recently, e.g. in *Heliconius* butterflies, where mate-choice behaviour is linked to a few major-effect genes [17].

The maintenance of combinations of complex traits is frequently controlled by supergenes, chromosomal regions with several tightly linked genes, controlling different traits and being protected from recombination [18,19]. Due to the tight linkage of individual genes in the supergene, the inheritance pattern of the encoded trait combination essentially follows that of a single-locus Mendelian trait [20]. This has, for example, been shown in the highly complex mating phenotypes of male plovers, distinguished by considerable behavioural and morphological differences [18]. Similarly, morphologically highly divergent male phenotypes in dwarf spiders are associated with a single supergene [20]. A supergene may also underlie abdominal constriction and somersault, both of which are needed to achieve monopolization of paternity and may be inherited in a linked fashion.

The fast evolution of mating traits in our species pair could also be explained via an association with the sex chromosomes. Widow spiders follow a simple sex determination system, with females carrying two diploid and males two haploid X chromosomes [21]. Several studies support the assumption that sexually selected traits share a large contribution of X-chromosomal loci [22,23], and X chromosomes are assumed to have faster evolutionary rates in relation to autosomes [24,25], owing to their smaller effective population size. While there is mixed evidence for this assumption, several examples from research in mammals and *Drosophila* flies lend support to this ‘large X-effect’ [26]. Especially genes associated with speciation appear to be located on X chromosomes [26].

Previous crossing experiments revealed that F1 hybrid males did not express the somersaulting behaviour of *L. hasselti*, which implies that it may be a recessive trait [14]. The haploid X-chromosomal state in males makes recessive alleles more responsive to selection [27]. The very recent divergence of the two species would also be consistent with the traits being encoded by faster evolving X-chromosomes [24,25]. However, there is no unequivocal evidence for sex-chromosome linkage of sexually selected traits across taxa [28]. Thus, even though sex-chromosome linkage may be possible for the observed traits in widow spiders, experimental evidence is still lacking.

**Figure 1.**
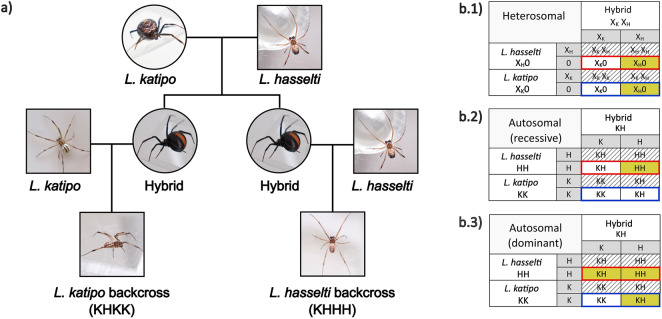
(a) Breeding scheme to produce backcross males and (b) possible inheritance patterns of self-sacrificial mating traits. (a) *Latrodectus katipo* females were crossed with *L. hasselti* males to generate F1 hybrid females, which were backcrossed to either *L. hasselti* males (producing KHHH) or *L. katipo* males (producing KHKK). Circles represent females, and squares represent males. (b) Possible inheritance patterns of the male mating traits assuming (b.1) an X-chromosomal origin, (b.2) an autosomal origin of recessive traits and (b.3) an autosomal origin of dominant traits. Red frames indicate genotypes of KHHH males, blue frames indicate those of KHKK males. Yellow cells represent the genotypes in which the trait expression is predicted. The photograph of *L. katipo* male was taken by Arnim Littek (Arnim on Inaturalist, CC BY 4.0). The background was digitally altered using AI-assisted image editing (Perplexity AI, March 2026) to enhance visual presentation; specimen morphology remained unaltered. Further photographs in panel (a) were taken by Miriam Scriba.

Considering the above background, we hypothesize that the contributing loci of the copulatory somersault and the abdominal constriction are tightly linked in a supergene on the X chromosome. Under this scenario, the trait set of somersaulting and abdominal constriction would have a simple Mendelian inheritance pattern. We tested these hypotheses by backcrossing the two species (figure 1a). Assuming the loci are tightly linked in a supergene, the somersaulting and constriction would always be co-inherited, with no recombination of the traits occurring. An origin of the mating traits on the haploid X chromosome would result in 50% of the males in each backcross group to express the recessive mating traits (figure 1b.1). In contrast, only 50% of the *L. hasselti* backcrosses, but none of the *L. katipo* backcrosses, would show the traits if they are recessive and of autosomal origin (figure1b.2). Lastly, if the traits are of autosomal dominant origin, all *L. hasselti* backcross males and 50% of the *L. katipo* backcross males would show the phenotype (figure1b.3).

## 2. Material and methods

### (a) Collection and rearing

*Latrodectus hasselti* egg sacs were collected from Cairnmuir and Alexandra, and *L. katipo* egg sacs from Kaitorete Spit, New Zealand and shipped to Hamburg. Egg sacs were kept at 24°C and 60% relative humidity in individual containers. Three to four weeks after hatching, spiderlings were moved to individual small vials (6.5 cm height × 2.5 cm diameter). All spiderlings were fed twice a week with three *Drosophila melanogaster*; and after the second moult, with three *D. hydei*. Following the fourth instar, females were moved to larger vials (9 cm height × 5 cm diameter) and fed either three maggots or two house flies.

### (b) Intraspecific crosses

Mating trials were conducted within the first two weeks after reaching sexual maturity. Adult males were introduced into female vials and removed the following day. Females were subsequently checked daily for the presence of an egg sac. Upon detection, egg sacs were weighed and stored in a climate chamber at 28°C and 50% relative humidity under a 12:12 h L:D cycle. Two to three weeks after hatching, spiderlings were transferred to individual vials and kept at 25 ± 2°C and 35% relative humidity under a 12:12 h L:D cycle. All spiders were fed twice a week and reared under the same conditions as their parents.

### (c) Interspecific crosses

Each *L. katipo* female was sequentially crossed with two sibling *L. hasselti* males to ensure a sufficient number of hybrids. This was necessary given that the fertility of the F1 generation is low [14]. Hybrids were reared under the same conditions as their parents. Upon reaching sexual maturity, each hybrid female was mated with either a *L. katipo* male to produce *L. katipo* backcrosses (KHKK) or with a *L. hasselti* male to produce *L. hasselti* backcrosses (KHHH) (figure 1a). Sexual cannibalism was frequently observed in mating trials with hybrid females.

### (d) Phenotyping of backcross male mating traits

Approximately one week after moulting to sexual maturity, each backcross male was paired with a *L. katipo* female. Only *L. katipo* females were used in these trials because they are not cannibalistic. Prior to the experiments, females were fed and placed on 3D-printed scaffolds for 3–4 days for web building, and males were fed 1 day before the experiments. Both males and females were weighed before the trials using an electronic balance (Mettler Toledo, accuracy: 0.0001g). Each trial was started by introducing a backcross male onto the female's web. Males generally initiate web reduction which reduces pheromone emission and attraction of rival males  [29]. Males that failed to initiate web reduction within 1–2 hours were replaced. Trials were terminated (i) immediately following two successful copulations, or (ii) after 5 hours in the case of only one or no successful mating. Mated spiders were stored at −72°C. Unmated males and females were tested again on the following days with different individuals. In total, 228 mating trials were conducted, of which 104 (*N*_KHHH_ = 55, *N*_KHKK_ = 49) resulted in successful mating. All experiments were recorded using Raspberry-Pi 4B units connected to RPi Camera NoIR modules (5MP resolution, 75° field of view, manually focused lens, two IR-LEDs).

**Figure 2.**
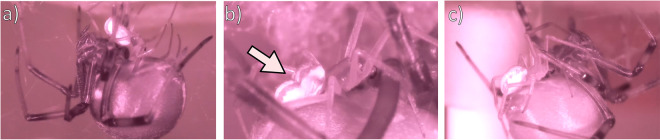
Images were captured from video footage of individual mating trials. (a) male performing somersault, (b) male displaying abdominal constriction, the arrow shows the site of constriction, (c) male displaying neither abdominal constriction nor somersault.

The presence or absence of abdominal constriction and somersault were scored during mating trials and subsequently verified by reviewing video recordings. A male was scored for the presence of somersault if it raised its abdomen and the fourth pair of legs upon insertion of the pedipalp (figure 2a), and abdominal constriction was identified by a visible narrowing of the mid-abdominal region with a constriction band (figure 2b). If a male did not attempt a somersault or did not develop an abdominal constriction (figure 2c), he was scored for the absence of the respective trait.

### (e) Statistical analyses

If somersaulting and abdominal constriction are linked at the same locus/supergene, we expect them to be always co-inherited. We hence tested whether the observed distribution of phenotypic linkage in the two backcross directions departed from 100% using a Fisher's exact test. Alternatively, they could be linked on the same chromosomes. Here, a recombination frequency below 50% is expected [30]. Departures from this distribution were tested with one-sample binomial tests.

To determine whether the expression of each trait deviated from 50% under the assumption of X-linkage, we tested the proportion of males performing somersault and the proportion of males displaying the abdominal constriction separately against a null expectation of 50% in each group (figure 1b.1), resulting in four one-sample binomial tests in total. In case of an autosomal recessive inheritance, we expect 50% of the *L. hasselti* backcross males and none of the *L. katipo* backcross males to show the traits (figure 1b.2). In case of an autosomal a dominant inheritance, we expect all *L. hasselti* backcross males and 50% of the *L. katipo* backcross males to show somersaulting and/or abdominal constriction (figure 1b.3). These expectations were tested with Fisher's exact tests.

Additionally, we analysed the effects of male and female body weights on each male behaviour (see the ESM).

## 3. Results

We phenotyped 104 F2 backcross males (*N*_KHHH_ = 55, *N*_KHKK_ = 49) for the presence of somersault and abdominal constriction during mating with *L. katipo* females. We used these data to test our two hypotheses, namely (i) that these two traits are tightly linked and (ii) that they have a simple Mendelian inheritance pattern, with contributing loci being X-linked.

### (a) Trait linkage

**Figure 3.**
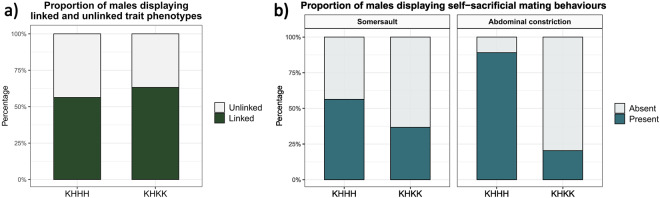
Proportion of the backcross males (*N*_KHHH_ = 55, *N*_KHKK_ = 49) (a) showing unlinked inheritance pattern (displaying either somersault or abdominal constriction) and linked inheritance pattern (expressing both traits or neither), (b) proportion of backcross males displaying self-sacrificial mating traits: copulatory somersault and abdominal constriction.

We predicted that these traits are linked and co-inherited, so that each individual should show either both of the traits or none of them. We found that the proportion of males displaying a phenotype of linked trait inheritance in both *L. hasselti* backcrosses (31/55) and *L. katipo* backcrosses (31/49) (figure 3a) significantly deviated from 100% (two-tailed Fisher's exact test, *p*_KHHH_ < 0.001, *p*_KHKK_ < 0.001) expected under tight linkage. In addition, recombination frequency between the two traits did not significantly depart from an expectation of 50% recombination frequency for each group (One-sample binomial test; *p*_KHHH_ = 0.419, *p*_KHKK_ = 0.085).

### (b) X-linkage

We predicted both traits to follow X-linked simple Mendelian inheritance, resulting in each trait being expressed in 50% of males in both backcrosses (figure 1b.1). The proportions of males displaying self-sacrificial somersault in both *L. hasselti* backcrosses (31/55) and *L. katipo* backcrosses (18/49) (figure 3b) were not significantly different from 50% (One-sample binomial test, *p*_KHHH_ = 0.419, *p*_KHKK_ = 0.085). In *L. hasselti* backcrosses, the phenotypic distribution of somersaulting deviated highly significantly from 100%, while in *L. katipo* backcrosses, it deviated highly significantly from 0% (Fisher's exact tests, *p*_KHHH_ < 0.001, *p*_KHKK_ < 0.001).

In contrast, the proportion of males with an abdominal constriction significantly deviated from 50% in both *L. hasselti* backcrosses (49/55) and *L. katipo* backcrosses (10/49; figure 3b) (One-sample binomial test: *p*_KHHH_ < 0.001, *p*_KHKK_ < 0.001). The majority of *L. hasselti* backcross males showed abdominal constriction during mating, whereas only a minority of *L. katipo* backcross males did so. The observed phenotypic distribution also significantly departs from 100% in *L. hasselti* backcross males (Fisher's exact test, *p*_KHHH_ < 0.05). In *L. katipo* backcross males, it significantly deviated from 0% (Fisher's exact test, *p*_KHKK_ < 0.01).

## 4. Discussion

The genetics of complex traits is still not well understood, especially behavioural characteristics, which have rarely been linked to genetic variation [31]. It was long assumed that such traits underlie a highly complex genetic basis with countless loci contributing to them [32,33]. However, recent work suggests that even complex behavioural traits can have a simple genetic basis, with a few loci or even only a single locus contributing to them [17]. Complex traits have also been linked to supergenes, tightly linked gene clusters which, despite containing multiple genes, behave like a single locus in inheritance [18,20]. A single supergene can encode various aspects of a complex trait. Making use of a very young sister species pair of widow spiders, distinguished by two complex mating traits, a stereotypical somersault to facilitate sexual cannibalism [10], and an abdominal constriction that prolongs male survival while being consumed by a female [13], we here explored the inheritance patterns of these traits using backcross experiments. Our results do not support the hypothesis that these two mating traits are tightly linked at a single locus/supergene, as both phenotypes freely recombine, with about half of the specimens in both backcrosses showing recombination of the traits. They must hence be encoded by two unlinked loci. The observed recombination frequencies suggest that the contributing loci are either very far apart on the same chromosome or even located on different chromosomes [30]. This is surprising, considering recent findings of highly complex mating traits in *Oedothorax gibbosus* spider being linked to a supergene [20].

We also hypothesized that the two traits are encoded by a single locus on an X-chromosome. Since our study species carry two X chromosomes [21], both traits could be encoded by one of them. Interestingly, somersaulting is very well explained by this inheritance pattern, supporting our hypothesis for this trait. Considering that somersaulting is a highly complex behavioural trait involving various movements and body positions, it may still be possible that the single X-chromosomal locus may comprise a supergene with multiple contributing linked genes [20]. An autosomal inheritance appears improbably for this trait, considering the results of our backcross experiments. It is quite remarkable that a highly complex phenotype such as somersaulting, which may lead to partial reproductive isolation, can have such a simple genetic basis. Our results are therefore relevant to speciation research. Speciation is often assumed to be achieved by the slow accumulation of genetic incompatibilities [34]. Our results show that unidirectional reproductive isolation can be achieved by a single X-linked locus. Note that *L. katipo* males cannot mate with *L. hasselti* females. The X-chromosomal location of this speciation locus is also well in line with the heterosomes being especially important carriers of speciation genes [35].

The expression rate of abdominal constriction significantly deviated from 50% in each backcross group. Therefore, its inheritance pattern is improbably to be explained by an X-chromosomal origin. Curiously, it also departs from the expectation of autosomal inheritance, suggesting that the genetic basis of the abdominal constriction is more complex. For example, the contributions of multiple loci to the trait expression could explain the departure from the expected phenotypic distributions under single-locus inheritance [36]. It should also be noted that the deviation from the expected phenotype in some males might be caused by variation in female traits that affect female chemical signalling (see supplementary material). Indeed, female pheromones are known to trigger male courtship behaviour as well as the abdominal constriction in *L. hasselti* males [13,37]. After all, genetic and environmental influences underlie most traits [38].

The evolutionary history of this fascinating sister-species pair points to a rapid loss of specialized mating traits in the New Zealand population. Despite the name ‘widow spiders’ for the entire genus, male sacrificial behaviour is observed in only 2 of its 31 species [10,39]. While one of them, *L. geometricus*, is phylogenetically very basal to the genus, the Australian *L. hasselti* is a rather modern species [16]. The male sacrificial phenotype was hence probably lost in most other *Latrodectus* species and reappeared in Australian *L. hasselti.* It is probably that New Zealand was colonized by *L. hasselti* from Australia, leading to the evolution of *L. katipo* approximately 100 000 years ago [16] and the loss of extreme mating traits. Our work highlights that a highly complex behavioural phenotype can have a simple genetic basis. Future genomic work on the two species and their crosses will help to identify the actual genes in the species’ genome linked to their highly divergent mating behaviour. Additional crossing experiments may also help narrow down the exact chromosomal location of the studied traits. Lastly, it would also be worthwhile to explore the genetic basis of the complementary females’ mating traits in these species. Like males, females of the sister species are also very different, with females of *L. hasselti* being larger and more aggressive than *L. katipo*. However, this is out of scope for this study, owing to the large amount of work required for phenotyping.

## Supplementary Material



## Data accessibility

Data associated with this manuscript are available from Dryad Digital Repository [40].
