# Supplementary material for: Crossing experiments suggest a simple genetic basis of complex male self-sacrifice phenotypes in widow spiders
Source: Biol Lett. 2026 Aug 12;22(8):20260211. doi: 10.1098/rsbl.2026.0211 (PMC13479370; doi:10.1098/rsbl.2026.0211)
Supplement: Supplementary file 1 [file rsbl.2026.0211.zip › rsbl.2026.0211/rsbl20260211_si_001.docx]

**Effect of male and female weight on copulatory somersault and abdominal constriction**

The copulatory somersault was shown by 31 out of 55 *Latrodectus hasselti* backcross males and 18 out of 49 *L. katipo* backcross males. The abdominal constriction was expressed by 49 out of 55 *L. hasselti* backcross males, and 10 out of 49 *L. katipo* backcross males.

We tested the effects of male and female body weight on the expression of male self-sacrificial mating behaviours by fitting a generalised linear model (GLM) with binomial distribution, including male behaviour as a response variable and male body weight and female body weight* as fixed effects. The models were run separately in R (v 4.4.2) [1] for each backcross type and each male behaviour, resulting in 4 separate models.

We found that male and female body weight had no effect on somersaulting behaviour in either backcross type, nor on abdominal constriction in *L. katipo* backcrosses (Table S1), while the likelihood of abdominal constriction increased with female weight in *L. hasselti* backcrosses (χ^2^_1_=5.83, p=0.016) (Table S1).

**Table S1. Model estimates of GLMs for the effects of male and female weights on the likelihood of expression of somersault and abdominal constriction. Significance is shown in bold. (KHHH: *L. hasselti* backcrosses, KHKK: *L. katipo* backcrosses)**

|  |  | Somersault | | | Abdominal constriction | | |
| --- | --- | --- | --- | --- | --- | --- | --- |
| Backcross type | Fixed effects | χ^2^ | df | p | χ^2^ | df | p |
| KHHH  (n=55) | male weight | 0.61 | 1 | 0.437 | 3.19 | 1 | 0.075 |
|  | female weight | 0.01 | 1 | 0.920 | **5.83** | **1** | **0.016** |
| KHKK  (n=49) | male weight | 0.16 | 1 | 0.693 | 0.35 | 1 | 0.556 |
|  | female weight | 0.32 | 1 | 0.572 | 0.92 | 1 | 0.339 |

**We compared the weight of females paired with each backcross group with a two-sample t-test and found no significant difference (t = 0.003, df = 102, p = 0.998; mean weight ± s.e. in mg; KHHH: 219.64 ± 6.86, n=55; KHKK: 219.61 ± 8.54, n=49).*

**Discussion**

We found no effect of female or male body weight on the expression of somersaulting behaviour supporting Forster`s suggestion that the copulatory somersault is a genetically programmed behaviour [2]. On the other hand, we found an effect of female weight on the abdominal constriction, but only in *L. hasselti* backcrosses. Female weight is positively correlated with fecundity in spiders [3] and males can assess female body condition via female pheromones [4,5]. In our study, the departure from expected Mendelian inheritance pattern and the female body weight effect in *L. hasselti* backcross males suggest that the expression of abdominal constriction is more complex and likely controlled by an interplay of multiple loci [6] and environmental factors. To understand the effects of environmental factors, future studies should investigate how female traits (such as sexual maturity, weight, age, and mating status) affect the expression of abdominal constriction in *L. hasselti* males as these traits may alter female chemical signalling [7–9] that triggers courtship behaviour as well as abdominal constriction in *L. hasselti* males [10].

**References**

1.	R Core Team. 2021 R: A Language and Environment for Statistical Computing.

2.	Forster L. 1992 The Stereotyped Behavior of Sexual Cannibalism in *Latrodectus hasselti* Thorell (Araneae, Theridiidae), the Australian Redback Spider. *Australian Journal of Zoology* **40**, 1–11. (doi:10.1071/ZO9920001)

3.	Higgins LE. 1992 Developmental plasticity and fecundity in the orb-weaving spider Nephila clavipes. *J. Arachnol* **20**, 94–106.

4.	MacLeod EC, Andrade MCB. 2014 Strong, convergent male mate choice along two preference axes in field populations of black widow spiders. *Animal Behaviour* **89**, 163–169. (doi:10.1016/j.anbehav.2013.12.023)

5.	Baruffaldi L, Andrade MCB. 2015 Contact pheromones mediate male preference in black widow spiders: avoidance of hungry sexual cannibals? *Animal Behaviour* **102**, 25–32. (doi:10.1016/j.anbehav.2015.01.007)

6.	Yi N, Xu S. 1999 Mapping quantitative trait loci for complex binary traits in outbred populations. *Heredity* **82**, 668–676. (doi:10.1046/j.1365-2540.1999.00529.x)

7.	Waner S, Motro U, Lubin Y, Harari AR. 2018 Male mate choice in a sexually cannibalistic widow spider. *Animal Behaviour* **137**, 189–196. (doi:10.1016/j.anbehav.2018.01.016)

8.	Sentenská L, Uhl G, Lubin Y. 2020 Alternative mating tactics in a cannibalistic widow spider: do males prefer the safer option? *Animal Behaviour* **160**, 53–59. (doi:10.1016/j.anbehav.2019.11.021)

9.	Stoltz JA, McNeil JN, Andrade MCB. 2007 Males assess chemical signals to discriminate just-mated females from virgins in redback spiders. *Animal Behaviour* **74**, 1669–1674. (doi:10.1016/j.anbehav.2007.03.011)

10.	Andrade MCB, Gu L, Stoltz JA. 2005 Novel male trait prolongs survival in suicidal mating. *Biol Lett* **1**, 276–279. (doi:10.1098/rsbl.2005.0318)
