# Supplementary material for: Crossing experiments suggest a simple genetic basis of complex male self-sacrifice phenotypes in widow spiders
Source: Biol Lett. 2026 Aug 12;22(8):20260211. doi: 10.1098/rsbl.2026.0211 (PMC13479370; doi:10.1098/rsbl.2026.0211)
Supplement: Supplementary file 1 [file rsbl.2026.0211.zip › rsbl.2026.0211/rsbl20260211_si_002.docx]

*Declaration of AI use*
The background of *L. katipo* male in Figure 1 was edited using AI tools to ensure visual consistency with other images in the same figure.

Date: March 2026
Model: Perplexity AI (Claude Sonnet)

Version: 4.6

Tool access: https://www.perplexity.ai

Prompt used: "Replace the background of the “image-1” with “image-2”. "

*Input*

Image-1

Image-2

*Output*
